# Association between high psychological distress and poor oral health-related quality of life (OHQoL) in Japanese community-dwelling people: the Nagasaki Islands Study

**DOI:** 10.1186/s12199-020-00919-9

**Published:** 2020-12-10

**Authors:** Ai Sekiguchi, Shin-ya Kawashiri, Hideaki Hayashida, Yuki Nagaura, Kenichi Nobusue, Fumiaki Nonaka, Hirotomo Yamanashi, Masayasu Kitamura, Koji Kawasaki, Hideki Fukuda, Takahiro Iwasaki, Toshiyuki Saito, Takahiro Maeda

**Affiliations:** 1 Department of Community Medicine, Nagasaki University Graduate School of Biomedical Sciences, 1-12-4 Sakamoto, Nagasaki, 852-8523 Japan; 2 Department of Oral Health, Nagasaki University Graduate School of Biomedical Sciences, Nagasaki, Japan; 3 Department of General Medicine, Nagasaki University Hospital, Nagasaki, Japan; 4 Department of Island and Community Medicine, Nagasaki University Graduate School of Biomedical Sciences, Nagasaki, Japan; 5 Community Medical Network Center, Nagasaki University Hospital, Nagasaki, Japan; 6 National Institute of Public Health, Saitama, Japan; 7 Department of Dentistry for the Disability and Oral Health, Division of Dysphagia Rehabilitation, Asahi University, Gifu, Japan

**Keywords:** General population, GOHAI, K6, Oral health, OHQoL, Periodontitis, Psychological distress

## Abstract

**Background:**

We investigated the association between psychological distress and oral health status/oral health-related quality of life (OHQoL) in Japanese community-dwelling people.

**Methods:**

We conducted a cross-sectional study using data from the Nagasaki Islands Study. A total of 1183 (455 men and 728 women) has been analyzed in this study. Psychological distress was measured using the Kessler Psychological Distress Scale (K6). Oral health status was measured by dental examination. The OHQoL was measured using the General Oral Health Assessment Index (GOHAI). We defined the total score of ≥5 points on the K6 as high psychological distress (high-K6 group).

**Results:**

The multiple linear regression analysis to identify the GOHAI showed that gender, K6, the total number of teeth, the number of dental caries, and visiting a dental clinic within the past 6 months significantly associated with the GOHAI. Among all of these variables, high-K6 (≥ 5) was a substantial contributing factor of the GOHAI (*β* = − 0.23, 95% Cl − 2.31 to −1.41, *p* < 0.0001).

**Conclusions:**

It is likely that the individual with high psychological distress was strongly related to poor OHQoL even in the general population.

## Background

The concern over mental health as an essential factor of the global burden of disease in public health has risen [1, 2]. Mental health is closely related to aspects of physical health such as cardiovascular disease [3] and diabetes [4] worldwide. In order to develop effective public health and clinical interventions for mental health, it is necessary to better understand the mechanisms that underlie interventions between mental health and other health conditions [5]. The association between mental health and oral health as a part of physical health has been examined [6, 7]. For example, patients with mental illnesses had a higher proportion of oral problems such as dental caries, periodontal disease, and edentulousness compared to a general population [8–10].

Kisely et al. [8, 9] suggested that oral health interventions for patients with mental illnesses should include advice on lifestyle habits and oral hygiene, the management of iatrogenic dry mouth, and early dental referral. It is important to develop and evaluate interventions that will increase the utilization of dental care (particularly preventive dental services) among people with mental illness in order to improve their oral health and reduce the dental expenditures among this vulnerable population [11].

Oral health-related quality of life (OHQoL), which is closely related to oral health status [12–16], is important for the management of oral health [17, 18]. Several studies reported the oral health status in patients with mental illnesses in psychiatric clinics [8, 9, 12, 13, 19], but few studies have investigated the oral health status of individuals in a general population who have or may have poor mental health. It has been proposed that public health interventions for psychological distress (which is not classified as mental illness) among individuals in general populations are important [20, 21].

Psychological distress was shown to be related to negative impacts on the number of teeth and oral health-related quality of life (OHQoL) in an older general population using self-reports [22]. A population-based cross-sectional study demonstrated that depression is associated with poor dental health evaluated by questionnaires [23]. The World Health Organization (WHO) recommended the approach of providing a program for the continuous improvement of oral health as an essential factor of general health that is related to quality of life [24]. The Japan Dental Association stated that good oral health is related to the extension of healthy life expectancy in Japan’s super-aging society [25]. Therefore, a clarification of the relationship between psychological distress and oral health status or OHQoL is a considerable task in public health. We hypothesized that psychological distress is closely related with poor oral health status as well as low OHQoL even in a general population. In the present study, we evaluated the participants’ oral health status by using questionnaires as well as dental examinations to clarify the precise associations between mental health status and oral health status/OHQoL in a general population.

We have conducted a community-based prospective cohort study (the Nagasaki Islands Study) that involves the results of an annual medical health check-up in Goto City, Nagasaki Prefecture, in the western part of Japan [26, 27]. The municipal government of Goto City has been promoting medical examinations of community-dwelling adults for screening and treating non-communicable diseases under Japan’s Ministry of Health, Labor and Welfare. As an additional investigation in the Nagasaki Islands Study, we evaluated the participants’ psychological distress and OHQoL by questionnaires and performed dental examinations.

The purpose of the present study was to clarify the association of psychological distress and oral health status with OHQoL in Japanese community-dwelling individuals, using data from the Nagasaki Islands Study.

## Participants and methods

### Study design and participants

This was a cross-sectional study as a part of the Nagasaki Islands Study. Data were collected to cover the whole region of Goto City in a 3-year cycle. A total of 3742 participants attended the annual medical health check-ups (1444 men and 2298 women) from 2015 to 2017; among them, we excluded the participants without all oral examinations (*n* = 2256), those whose measuring probing pocket depth (PPD) and clinical attachment loss (CAL) were not measured (*n* = 270), those without General Oral Health Assessment Index (GOHAI) scores (*n* = 24), and those who underwent health check-ups more than two times in the 3-year period (*n* = 9). The final total number of participants in this study was thus 1183 (455 men and 728 women) (1183 of 3742, 32%). This study was approved by the Ethics Committee of Nagasaki University Graduate School of Biomedical Sciences (study registration number 14051404-8) and conformed to the Declaration of Helsinki. Written informed consent forms were provided in Japanese to ensure comprehensive understanding of the study objectives, and informed consent was given by all participants.

### Measures

#### Demographic variables

The following demographic variables were evaluated: age; sex; body mass index (BMI); a past medical history of stroke, ischemic heart disease, hypertension, diabetes mellitus, or dyslipidemia (under management/no history); falling within the past 1 year; hospitalization within the past 1 year (yes/no); smoking status (yes/no); habitual drinking (yes/few); exercise activity [exercise for 30 min or more at least twice a week for 1 year or more] (yes/no), social participation with neighbors [having neighbors who can have a close conversation] (yes/no), social participation with non-neighbors [have friends, separated family members, and relatives who can come and go intimately] (yes/no), and having any hobbies (yes/no). All demographic variables except for age and sex were collected through interviews with trained researchers at each of the city’s community centers.

#### Psychological distress

We used the Kessler Psychological Distress 6-question Short-Form Scale (K6) to measure the participants’ psychological distress [28]. The K6 is a questionnaire developed to examine the respondent’s general psychological distress in a community setting. It consists of the following six items: “During the last 30 days, how often did you feel (1) nervous, (2) hopeless, (3) so restless or fidgety, (4) so depressed that nothing could cheer you up, (5) that everything was an effort, (6) worthless?” Each of these items is answered using the following 5-point scale: all of the time (4 points), most of the time (3 points), some of the time (2 points), a little of the time (1 point), never (0 points). The total score ranges from 0 to 24, and higher scores indicate more severe psychological distress or probable mental illness. The K6 was translated into Japanese, and the validity of the Japanese version of the K6 was confirmed [29, 30]. In previous study using K6 in Japan, the total score of ≥ 5 points and ≥ 13 points on the K6 were defined as moderate and severe psychological distress, respectively [20, 21, 31]. To detect psychological distress widely in a general population, we defined the total score of ≥ 5 points on the K6 as high psychological distress. The K6 was administered during interviews with trained researchers at each community center.

#### Dental examinations

We investigated the participants’ oral health status based on the following parameters: the number of teeth, the number of untreated dental caries, the PPD, the CAL, the periodontal stage (early/advanced) (yes/no), and whether the participant’s most recent dental visit was within the prior 6 months (yes/no). A periodontal examination was performed using a method that is a modification of the Third National Health and Nutrition Examination Survey [32] by one of four trained dentists, as described [33]. The PPD was defined as the distance from the gingival margin to the bottom of the pocket, and the CAL was defined as the distance from the cemento-enamel junction to the bottom of the pocket. These values were measured using a periodontal probe at the mesio-buccal and mid-buccal sites for all present teeth excluding the third molars.

In these oral examinations, the periodontal stage, PPD, and CAL were measured in the participants who had ≥ 10 teeth. The periodontal stage was defined as follows: the worst PPD (i.e., 4–5 mm) was defined as early-stage, and a PPD ≥ 6 mm for at least one tooth among the present teeth was defined as advanced-stage. The averages of the PPD and CAL were calculated as the sum of PPD and the sum of the CAL divided by the participant’s total number of teeth, as the mean PPD (mPPD) and the mean CAL (mCAL), respectively. Prior to the start of this study, all examiners were trained with the use of charts, periodontal models, and volunteer participants at the Nagasaki University Hospital.

#### OHQoL

We used the General Oral Health Assessment Index (GOHAI) [34] to measure each participant’s OHQoL. The GOHAI is a questionnaire that was developed specifically to evaluate the OHQoL. It contains the following 12 items: “During the last 3 months, how often (1) did you limit the kinds or amounts of food you eat because of problems with your teeth or dentures, (2) did you have trouble biting or chewing any kinds of food, such as firm meat or apples, (3) were you able to swallow comfortably, (4) have your teeth or dentures prevented you from speaking the way you wanted, (5) were you able to eat anything without feeling discomfort, (6) did you limit contacts with people because of the condition of your teeth or dentures, (7) were you pleased or happy with the looks of your teeth and gums or dentures, (8) did you use medication to relieve pain or discomfort around your mouth, (9) were you worried or concerned about the problems with your teeth, gums, or dentures, (10) did you feel nervous or self-conscious because of problems with your teeth, gums, or dentures, (11) did you feel uncomfortable eating in front of people because of problems with your teeth, gums, or dentures, and (12) were your teeth or gums sensitive to hot, cold, or sweets?” The GOHAI was translated into Japanese, and the validity of the Japanese version was confirmed [35, 36].

While the original version of the GOHAI included three positively worded questions, i.e., questions (3), (5), and (7), the GOHAI Japanese version was changed to include only negatively worded questions for usability [35]. Each of the 12 items has a score between 1 and 5: all of the time (1 point), most of the time (2 points), some of the time (3 points), a little of the time (4 points), and never (5 points). Although there is no global standard cutoff point for the GOHAI, the cumulative score is from 12 to 60 points, and a lower score indicates poorer OHQoL. The GOHAI was administered during interviews by trained researchers after dental examinations at each community center.

#### Statistical analyses

We first performed a descriptive analysis. We then divided all of the participants into two groups based on their psychological distress status revealed by the K6: those whose score was high (≥ 5 points) and those whose K6 score was low (< 5 points). We then used χ^2^ tests to compare the ratio of categorical variables and *t* tests to compare the averages of continuous variables between the high and low K6 group.

We conducted a correlation analysis of the GOHAI in continuous variables among dental examinations and performed *t* tests to compare the averages of GOHAI scores between two dental variables. Last, we performed a multiple regression analysis to identify factors related to the GOHAI scores. In these statistical analyses, missing values were excluded. We attempted to identify any variables that independently contribute to GOHAI from the variables [gender, K6, habitually exercising, social participation with neighbors, falling within 1 year, hospitalized within 1 year, total number of teeth, number of dental caries, mCAL, visit a dental within 6 months] that were significantly associated with the GOHAI score by performing a multiple regression analysis. All statistical analyses were carried out with two-sided tests. The threshold for significance was *p* < 0.05. The analyses were conducted using JMP® 14 (SAS Institute, Cary, NC, USA).

## Results

### Demographic variables

The demographic variables of the participants are summarized in Table 1. The mean (SD) age was 65.9 (11.7) years old; 62% of the participants (*n* = 728) were female. Nineteen percent of the participants (*n* = 221) reported having fallen within the past 1 year, and 124 (10%) experienced hospitalization within the past year. Regarding activities, 581 participants (49%) habitually exercised, 936 participants (79%) had social participation with neighbors, 1065 participants (90%) had social participation with non-neighbors, and 975 participants (82%) had a hobby.

**Table 1:** Comparisons of demographic variables between the K6 high group and the K6 low group

	Participants	Psychological distress status		
	Total	High K6 (≥ 5)	Low K6 (< 5)	***p*** value
	(***n*** = 1183)	(***n*** = 125)	(***n*** = 1058)	
Age, mean (SD)	65.9 (11.7)	64.7 (14.0)	66.1 (11.3)	0.20
Female, *n* (%)	728 (62)	91 (73)	637 (60)	0.006*
BMI, mean (SD)	22.9 (3.2)	22.5 (3.1)	22.9 (3.3)	0.12
Past medical history of, *n* (%)				
Stroke	37 (3)	4 (3)	33 (3)	0.96
Ischemic heart disease	63 (5)	8 (6)	55 (5)	0.57
Hypertension	413 (35)	38 (30)	375 (35)	0.26
Diabetes mellitus	72 (6)	7 (6)	65 (6)	0.81
Dyslipidemia	236 (20)	27 (22)	209 (20)	0.63
Falling within 1 year	221 (19)	41 (33)	180 (17)	< 0.0001^†^
Hospitalization within 1 year	124 (10)	22 (18)	102 (10)	0.006*
Current smoking, *n* (%)	99 (8)	12 (9)	87 (8)	0.60
Habitual drinking, *n* (%)	358 (30)	29 (23)	329 (31)	0.068
Activities, *n* (%)				
Habitual exercising^a^	581 (49)	40 (32)	541 (51)	< 0.0001^†^
Social participation with neighbors	936 (79)	77 (62)	859 (81)	< 0.0001^†^
Social participation with non-neighbors	1065 (90)	107 (86)	958 (91)	0.081
Having any hobbies	975 (82)	94 (75)	881 (83)	0.025*

Chi-squared tests were used to compare the ratio of categorical variables, and *t* tests were used to compare the averages of continuous variables between the groups. Missing value excluded; falling within 1 year = 5, habitual drinking = 1, social participation with neighbors = 1, the other variables were not missing value

*BMI* body mass index, *K6* the Kessler Psychological Distress 6-Question Short-Form Scale

*p < 0.05

^†^*p* < 0.001

^a^Exercise of ≥ 30 min, ≥ 2×/week, and ≥ 1 year

### Comparisons of the high- and low-K6 groups’ demographic variables

There were no significant between-group differences in age, BMI, any aspect of past medical history, smoking status, habitual drinking, or social participation with non-neighbors. The mean (SD) of the cumulative K6 score for all participants was 1.5 (2.9) points. The percentage of participants with a high K6 score (≥ 5) was 125 (10.6%); that of the low-K6 group was 89.4% (*n* = 1058). The distribution of K6 scores is illustrated in Supplemental Figure S1.

The results of our comparison of demographic variables between the high-K6 and low-K6 groups are given in Table 1. The percentage of women was significantly higher in the high-K6 group (*p* = 0.006). Compared to the low-K6 group, the percentages of participants who reported having fallen within the past year (*p* < 0.0001) and those who had been hospitalized within the past year (*p* = 0.006) were significantly higher in the high-K6 group. Compared to the low-K6 group, the high-K6 group’s percentages of participants who habitually exercised (*p* < 0.0001), engaged in social activity with neighbors (*p* < 0.0001), and had any hobbies (*p* = 0.025) were significantly lower.

### Comparisons of the high- and low-K6 groups’ oral health status

The oral health status data are summarized in Table 2. For the total population, the mean (SD) total number of teeth was 23.3 (5.1), the mean (SD) number of untreated dental caries was 0.7 (1.7), the mean (SD) mPPD was 2.0 (0.6), the mean (SD) mCAL was 2.4 (0.9), and the mean (SD) GOHAI score was 55.9 (5.0). Regarding periodontal stages, 348 participants (29%) were at the early periodontal stage and 246 participants (21%) were at the advanced periodontal stage. Five hundred nine participants (43%) reported having visited a dental clinic within the past 6 months.

**Table 2:** Comparisons of oral health status between the K6 high group and the K6 low group

	Participants	Psychological distress status		
	Total	High K6 (≥ 5)	Low K6 (< 5)	***p*** value
	(***n*** = 1183)	(***n*** = 125)	(***n*** = 1058)	
Total no. of teeth, mean (SD)	23.3 (5.1)	23.0 (0.5)	23.4 (0.2)	0.46
No. of untreated dental caries, mean (SD)	0.7 (1.7)	0.7 (0.2)	0.6 (0.1)	0.60
mPPD (mm), mean (SD)	2.0 (0.6)	2.1 (0.7)	2.0 (0.6)	0.16
mCAL (mm), mean (SD)	2.4 (0.9)	2.6 (1.1)	2.4 (0.8)	0.053
Periodontal stages, *n* (%)				
Early stage (PPD = 4–5 mm)	348 (29)	37 (30)	311 (29)	0.96
Advanced stage (PPD ≥ 6 mm)	246 (21)	26 (21)	220 (21)	0.998
Visit a dental within 6 months, *n* (%)	509 (43)	47 (38)	462 (44)	0.195
GOHAI, mean (SD)	55.9 (5.0)	52.3 (7.6)	56.4 (4.4)	< 0.0001^†^

Chi-squared tests were used to compare the ratio of categorical variables, and *t* tests were used to compare the averages of continuous variables between the groups

*mPPD* mean probing pocket depth, *mCAL* mean clinical attachment loss, *GOHAI* the General Oral Health Assessment Index

*p < 0.05

†*p* < 0.001

We compared the oral health status between the high- and low-K6 groups (Table 2). The GOHAI scores were significantly lower in the high-K6 group compared to the low-K6 group (*p* < 0.0001). The mPPD and the mCAL values tended to be higher in the high-K6 group compared to the low-K6 group. There was a tendency for fewer participants having visited a dental clinic within the past 6 months in the high-K6 group compared to the low-K6 group.

### Comparison of GOHAI scores between dental examinations

Each of the following was significantly correlated with the GOHAI scores: the total number of teeth (*r* = 0.17, *p* < 0.0001), the number of dental caries (*r* = − 0.14, *p* < 0.0001), and the mCAL (*r* = − 0.09, *p* = 0.003). The mPPD tended to be correlated with the GOHAI (*r* = − 0.04, *p* = 0.16). The GOHAI scores were significantly lower in the participants who had visited a dental clinic within the previous 6 months compared to those who had not (mean; yes vs. no, 55.6 vs. 56.2, *p* = 0.025). The GOHAI scores tended to be lower in the participants with advanced periodontal disease compared to those without it (55.4 vs. 56.1, *p* = 0.06).

### Multiple regression analysis to determine the factors identifying the GOHAI

Table 3 lists the results of the multiple regression analysis performed to determine the factors identifying the GOHAI, adjusted with the following co-efficient variables: gender, K6 score, habitual exercising, social participation with neighbors, falling within the past year, hospitalization within the past year, the total number of teeth, the number of dental caries, the mCAL, and visiting a dental clinic within the past 6 months. A significant regression equation was found: *F* (10, 1166) = 16.60, *p* < 0.0001, with *R*^2^ = 0.12, root-mean-square error (RMSE) = 4.68, variance inflation factor (VIF) = 1.03–1.25. The results of the multiple regression analysis showed that gender, K6, the total number of teeth, the number of dental caries, and visiting a dental clinic within the past 6 months were significant contributing factors of the GOHAI. Among all of these variables, high-K6 was a substantial contributing factor of the GOHAI, independently (*β* = − 0.23, *t* = − 8.12, *p* < 0.0001).

**Table 3:** Multiple regression analysis to determine the factors identifying GOHAI

Independent variables	***β***	95%CI	***p*** value
Male gender	0.10	0.24–0.81	0.0003^†^
K6 (≥5)	− 0.23	− 2.31 to − 1.41	< 0.0001^†^
Habitual exercising ^a^ (yes)	0.05	− 0.02–0.53	0.07
Social participation with neighbors (no)	0.02	− 0.23–0.45	0.51
Falling within 1 year (no)	0.04	− 0.08–0.62	0.13
Hospitalization within 1 year (no)	0.02	− 0.29–0.59	0.51
Total number of teeth	0.11	0.05–0.17	0.0004^†^
Number of dental caries	− 0.13	− 0.55 to − 0.23	< 0.0001^†^
mCAL	− 0.05	− 0.64–0.04	0.09
Visit a dental within 6 months (yes)	− 0.07	− 0.63 to − 0.08	0.01*

Missing value excluded; falling within 1 year = 5, social participation with neighbors = 1, total analyzed participants = 1177 out of 1183

*p < 0.05

^†^*p* < 0.001

^a^Exercise of ≥ 30 min, ≥ 2×/week and ≥ 1 year

## Discussion

This cross-sectional study aimed to clarify the association of psychological distress and oral health status with OHQoL in Japanese community-dwelling individuals by using data from the Nagasaki Islands Study. The results of our analyses demonstrated that psychological distress as defined by a K6 score ≥ 5 points and OHQoL defined by the GOHAI were strongly related in this community-dwelling population.

We observed that high psychological distress reflected by the K6 score was associated with female gender, the lack of habitual exercise, no social participation with neighbors, falling within the prior year, and low OHQoL as shown by the GOHAI. In previous studies, female gender, falling, and poor social participation were also related to psychological distress [37–40]. The new knowledge gained in the present study is that low OHQoL was closely related to psychological distress in a community setting.

Poor mental health status has been reported to be associated with both poor oral health status and low OHQoL [12, 13]. A population with mental illness was reported to be likely to have more risk factors related to poor oral health (e.g., lack of motivation to engage in oral hygiene behaviors and adverse effects of medications [mainly xerostomia]) compared to the general population [10]. Our present findings revealed that the lack of social participation with neighbors was strongly related to the high psychological distress indexed by the K6. This result is compatible with a study that showed a positive association between social participation and mental and physical health [39].

Moreover, the mPPD and the mCAL values in the present study tended to be higher in the high-K6 group compared to the low-K6 group, although the differences were not significant. We have speculated that high psychological distress may be associated with a reduction of daily activities such as both social participation and oral hygiene behaviors in community settings. Previous studies showed that oral health problems including periodontitis may play a role in the development or worsening of depressive symptoms [41, 42]. Considering our present findings, it appears that clinicians should pay attention to their patients’ psychogenic background in the management of oral health care and OHQoL for the optimal oral health care of individuals with high psychological distress, even in the general population.

The results of our analyses demonstrated that the factors contributing to poor OHQoL as shown by the GOHAI were female gender, high psychological distress reflected by the K6, the total number of teeth, the number of dental caries, and having visited a dental clinic within the previous 6 months. Unsurprisingly, dental conditions are closely correlated with the OHQoL scores [12–14, 16]. In the present study, notably, psychological distress as revealed by the K6 was a substantial contributing factor to OHQoL. The previous reports proposed that the patient’s psychogenic background should be considered as a factor of patient’s subjective symptoms, such as medically unexplained toothache, atypical odontalgia, or self-reported xerostomia, that may be associated with reduced OHQoL in the management of oral care [43, 44]. The results of the present study suggested that people with high psychological distress may perceive subjectively OHQoL as poor in a general population. On the other hand, it has been reported that compared to individuals without psychological distress, those with poor mental health are likely to use oral health services less often [19]. In the present study, the percentage of participants who had visited a dental clinic within the past 6 months tended to be lower in the high-K6 group compared to the low-K6 group (although the difference was not significant), and this suggests that individuals with high psychological distress may not be likely to visit a dental clinic even if they have poor OHQoL. Although the participants in the present study had mild both psychological distress and oral health problems, high psychological distress strongly related to poor OHQoL even in community-dwelling people.

The World Health Organization (WHO) recommended the approach of providing a program for the continuous improvement of oral health as an essential factor of general health that is related to quality of life [24]. The Japan Dental Association [25] stated that good oral health is related to the extension of healthy life expectancy in Japan’s super-aging society. People who make regular dental visits are at a low risk of tooth loss [45]. A randomized controlled study demonstrated that an oral health promotion program for individuals with mental illness reduced their plaque index values [46]. We suggest that it is important to encourage individuals with high psychological distress (even those in general populations) to have regular dental health visits not only for the treatment of caries or periodontal disease but also for the maintenance of their oral health to prevent the deterioration of their OHQoL.

Another interesting finding of the present study is that compared to the men, the women had higher psychological distress and poorer OHQoL. During their lifetime, women worldwide are about twice as likely as men to develop depression [47]. In global and long-term trends, the relationship between female gender and the risk of mental health may be explained by a previously reported gender gap [37]. In addition, although women have reported more complaints about dental pain and chewing problems and showed greater concern about their oral health’s social and psychological impacts than men, these gender differences vary among distinct populations [48]. A consideration of the gender gap may thus be important when seeking to maintain the appropriate psychological distress levels and oral health management in general populations.

There are some potential study limitations that warrant discussion. First, because it was a cross-sectional study, a causal relationship was not shown. Longitudinal studies are necessary to clarify causality in the future. Second, the mean age of the participants was somewhat elderly at 65.9 years, and it is possible that our findings cannot be extrapolated to younger populations. Because it has been reported that the proportion of high psychological distress is higher in not only older populations but also younger populations [20], additional research is necessary to clarify the association between psychological distress and oral health status in younger populations.

Third, the ratio of individuals undergoing oral health examinations in their annual health check-ups was < 50% of the participants. Our results may not reflect the status of the overall health check-up population. Fourth, because the K6 is not designed for the diagnosis of mental illnesses and we did not ask the participants about their history of using psychotropic drugs, it was difficult to identify whether or not the participants had mental illnesses. Fifth, some selection biases might have been present because we did not obtain the status of psychological distress and oral health in individuals who did not undergo the health check-ups. The frequency of complications such as stroke, ischemic heart disease, and diabetes mellitus that could affect psychological distress was low in the present study, but comparable to another Japanese cohort study [49]. Lastly, we could not include all coefficient factors, such as education level [50] and economic status [51], which were reported as important factors for preventive care in public health.

## Conclusion

This is the first study to clarify the significant association between high psychological distress and poor OHQoL in a Japanese community-dwelling population. It is likely that the individual with high psychological distress was strongly related to poor OHQoL even in the general population.

## Supplementary Information

**Additional file 1:** **Suppl. Fig. S1**. Distribution of K6.

## Data Availability

The datasets used and/or analyzed during the present study are available from the corresponding author on reasonable request.

## Abbreviations

CAL: Clinical attachment loss
GOHAI: General Oral Health Assessment Index
K6: Kessler Psychological Distress 6-Question Short-Form Scale
mPPD: Mean probing pocket depth
mCAL: Mean clinical attachment loss
OHQoL: Oral health-related quality of life
PPD: Probing pocket depth
