# Supplementary figures and images for: Association between high psychological distress and poor oral health-related quality of life (OHQoL) in Japanese community-dwelling people: the Nagasaki Islands Study
Source: Environ Health Prev Med. 2020 Dec 10;25:82. doi: 10.1186/s12199-020-00919-9 (PMC7730733; doi:10.1186/s12199-020-00919-9)

## Slide 1
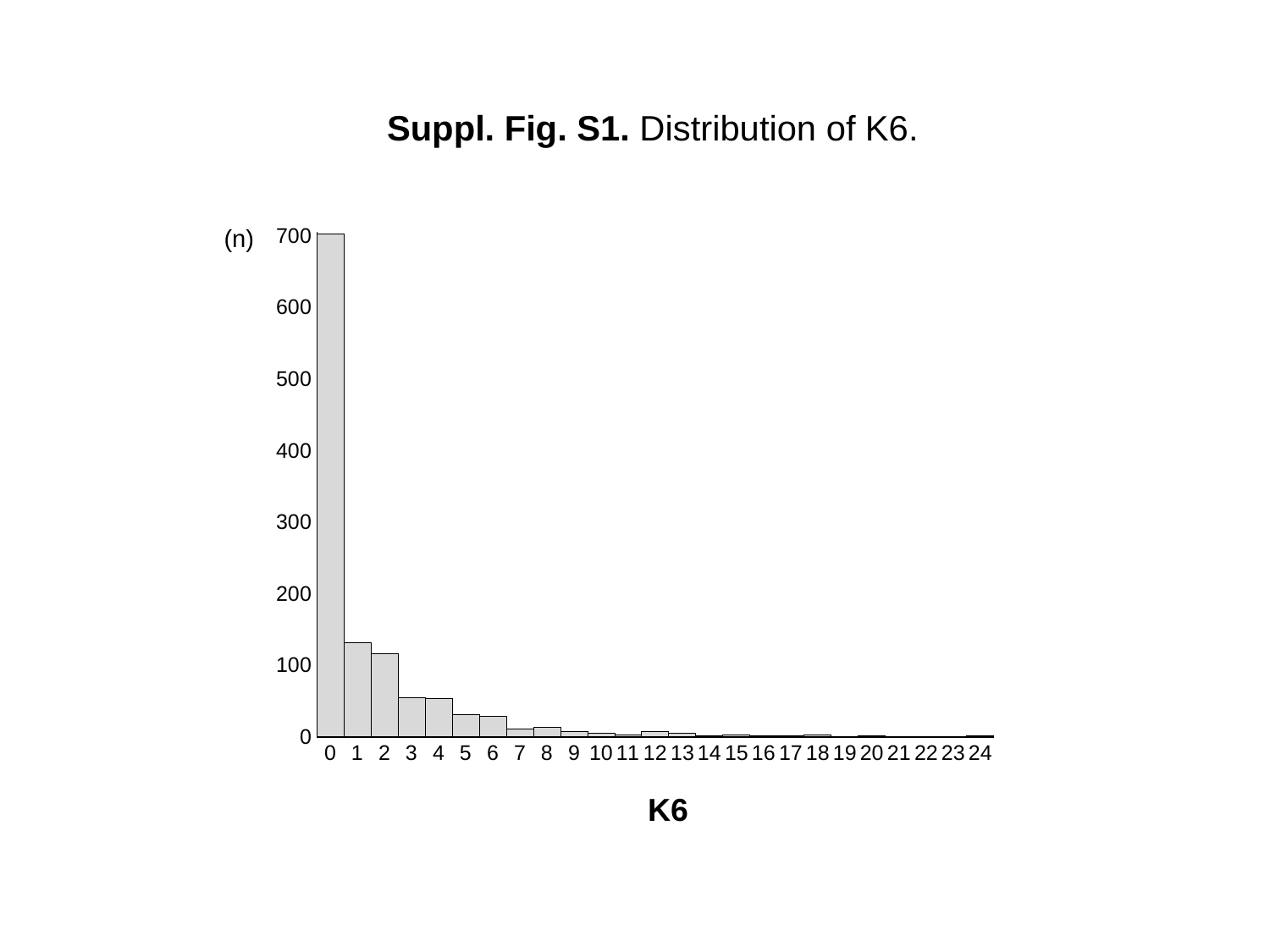

Suppl. Fig. S1. Distribution of K6.
### Chart
| Category | |
|---|---|
| 0 | 702.0 |
| 1 | 131.0 |
| 2 | 116.0 |
| 3 | 55.0 |
| 4 | 54.0 |
| 5 | 31.0 |
| 6 | 29.0 |
| 7 | 11.0 |
| 8 | 13.0 |
| 9 | 7.0 |
| 10 | 5.0 |
| 11 | 3.0 |
| 12 | 8.0 |
| 13 | 5.0 |
| 14 | 1.0 |
| 15 | 3.0 |
| 16 | 1.0 |
| 17 | 2.0 |
| 18 | 3.0 |
| 19 | 0.0 |
| 20 | 1.0 |
| 21 | 0.0 |
| 22 | 0.0 |
| 23 | 0.0 |
| 24 | 2.0 |(n)
K6

Supplement: Supplementary file 1 — Additional file 1: Suppl. Fig. S1. Distribution of K6. [file 12199_2020_919_MOESM1_ESM.pptx]
